# Work, family and social environment in patients with Fibromyalgia in Spain: an epidemiological study: EPIFFAC study

**DOI:** 10.1186/s12913-014-0513-5

**Published:** 2014-11-11

**Authors:** Antonio Collado, Emili Gomez, Rosa Coscolla, Ruth Sunyol, Emília Solé, Javier Rivera, Emília Altarriba, Jordi Carbonell, Xavier Castells

**Affiliations:** Unidad de Fibromialgia, ICEMEQ, Hospital Clínic de Barcelona, C/ Villarroel 170, Barcelona, 08036 Spain; Unidad de Fibromialgia, Hospital del Mar, Pg Marítim 25-29, Barcelona, 08003 Spain; Unidad de Fibromialgia, Hospital General Universitario Gregorio Marañón, C/ Doctor Esquerdo 46, Madrid, 28007 Spain; Fundación Pere Tarrés, C/ de les Carolines 10, Barcelona, 08012 Spain; Fundación FF, Avd/ Diagonal 365 4°1ª, Barcelona, 08037 Spain; Servicio de Epidemiología y Evaluación, Hospital del Mar, Pg Marítim 25-29, Barcelona, 08003 Spain

**Keywords:** Fibromyalgia, Family, Work, Social impact

## Abstract

**Background:**

Fibromyalgia (FM) is a condition characterized by widespread pain, estimated to affect 2.4% of the Spanish population. Nowadays, there are no consistent epidemiological studies on the actual impact of the disease on work and family of these patients in a representative manner; therefore, the purpose of the study is to analyze the impact on family, employment and social environment in a representative sample of patients with FM attending Primary Public Care Centers in Spain.

**Methods:**

We carried out an epidemiological study, with a probability sampling procedure, stratified, relative to the municipality size and the number of health centres, seeking territorial representation. The survey was conducted using a self-administered structured questionnaire.

**Results:**

A sample of 325 patients with FM was studied in 35 Primary Health Care Centers (PHCCs). The sample is composed of 96.6% of women, 51.9 (8) years of mean (standard deviation- sd) age. Ninety-three percent of the patients have worked throughout their life. Mean (sd) age onset of symptoms was 37 (11) years and diagnosis of FM was established 6.6 (8) years later.

*Family Environment*: Fifty-nine percent of patients have difficulties with their partner. Forty-four percent of the patients report to be fairly or totally dependent on a family member in household chores. The household income decreased a mean (sd) of 708 (504) Euros/month in 65% of the patients. In 81% of the patients, there was an increase in extra expenses related to the disease with a mean (sd) of 230 (192) Euros/month.

*Working environment*: At the moment of the study, 45% of the patients had work activity (34% were working and 11% were at sick leave), 13% were unemployed seeking job and 42% were not in the labor force. Twenty-three percent of patients had some degree of permanent work disability pension.

*Social Environment*: The degree of satisfaction with health care professionals was low and twenty-six percent of the patients were members of specific patients associations.

**Conclusions:**

This study finds that people with FM who visit PHCCs of Spain experience a high impact on families and employment with heavy loss of ability to work.

## Background

Fibromyalgia (FM) is a chronic disease that affects 2.4% of the Spanish general population [1], a percentage similar to that observed in different European countries [2]. The main symptoms are chronic widespread pain, fatigue, and other emotional and cognitive symptoms that significantly affect the quality of life of patients [3]. Some studies performed in several countries have reported a major impact of the disease on ability to work [4-7] as well as family and social relations [8].

In Spain, some clinical studies have also observed that FM patients experience a varied impact on their ability to work. Between 43% and 78% of patients with FM are in sick leave, and the total disability status ranges between 6.7% and 30% [9-13]. Moreover, there haven’t been studies conducted in Spain on the state of family relationship in patients with Fibromyalgia.

The observed differences regarding the employment status, might be related to the origin of the patients studied, the sample size and the criteria used in the selection of the patients. As a matter of fact, there are no consistent epidemiological studies that show us the actual influence of the disease on work and family of these patients in a representative manner.

On the other hand, significant details of the ability to work and family impact, such as the quantity of job changes, job losses, the degree of family disruption, associated family burdens, family economic losses, and administrative or social response, still remain unknown. These aspects are collected in the International Classification of Functioning, Disability and Health (ICF) [14]. The ICF is described as the complex interplay of the health components body functions, body structures, activities and participation and contextual factors, such as environmental and personal factors. The questionnaires commonly used to measure the impact of the disease in patients with FM, such as Fibromyalgia Impact Questionnaire (F.I.Q) and others, specially include concepts linked to body functions and fewer are linked to activities and participation or environmental factors [15]. For this reason we have designed a self-administered questionnaire that collects more extensive the influence that the disease has on the patient’s activities and environmental factors, and obtain representative data on the situation in Spain. This research was conducted with the objective of doing an epidemiological study of the consequences and the responses generated in the work, family, social and administrative environments, in a representative sample population diagnosed with FM attending Primary Health Care within the Public Health System.

## Methods

Patients with a diagnosis of FM, codified with the code M79.7 according to the International Classification of Diseases (ICD-10), aged between 16 and 64 years (age range for working-age person in Spain) attending Public Health Centers in Spain, were studied. The patients were previously diagnosed of FM by family physicians and/or specialists in Rheumatology, following the American College of Rheumatology 1990 Criteria [16].

Exclusion criteria were not having an adequate cognitive ability to answer the evaluation questionnaire and/or no signing informed consent.

### Sampling of the PHCCs

It was proposed a probability sampling procedure, with poly-staged, stratified cluster sampling. The strata were defined by the size of the Spanish municipalities, divided into 3 categories: Stratum I (municipalities up to 20,000 inhabitants), Stratum II (municipalities between 20,001 and 100,000 inhabitants) and Stratum III (municipalities with more than 100,000 inhabitants).

The sample was defined according to the following stages: size of municipality, Primary Health Care Center (PHCC), and patients. It was sought a regional representation according to different Autonomous Communities (AC) of Spain, excluding Ceuta and Melilla because of their extreme population differences.

With the aim of getting the most representative sample possible, we select the patients of the PHCCs instead of specialized hospital units or FM patients associations in order to minimize selection bias. To make the particular selection of the PHCCs, it was required that the 17 AC were represented in each of the three strata by, at least, one PHCC, except in the most populated AC - Andalucía, Catalunya and the Comunidad de Madrid, where two PHCCs were assigned in Stratum I.

From the general list of 2,980 PHCCs included in the Catalog of Primary Care Centers of the National Health System, − on a completely random basis - 54 PHCCs: 20 in Stratum I, 17 in Stratum II, and 17 in Stratum III were selected. In addition, we selected 44 alternate PHCCs. From a total of 98 PHCCs, 52 agreed to participate in the study (acceptance rate 53,1%). In three AC (Valencia, País Vasco and La Rioja), it was not possible to involve any center, and no response was received from 17 PHCCs. Finally, a total of 35 PHCCs participated in the study.

**Figure 1:**
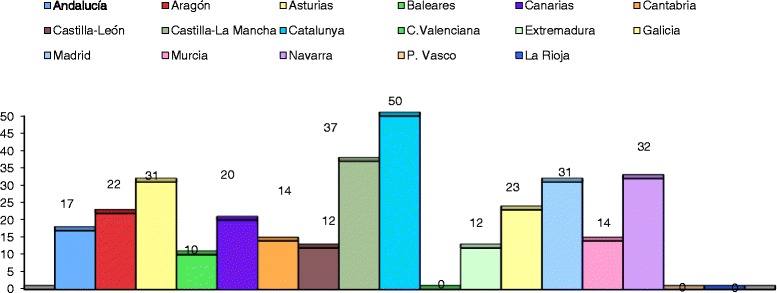
**Distribution of patients by Autonomous Communities.** The number of patients included in the study distributed by different Autonomous Communities in Spain.

Among the participating PHCCs, 11 were from Stratum I, 11 were from Stratum II, and 13 were from Stratum III, distributed among 14 AC (Figure 1). The final rate of participation of PHCCs was 35,7%, with a possible population of 530 people.

### Sample size

From each PHCC, there was obtained a maximum number of consecutively examined patients: 10 in Stratum I and 20 patients in Stratum II and III. Of the 530 questionnaires sent out, 375 were completed. Of these 375 questionnaires, 50 were excluded (28 were unanswered or with a low level of responses - twenty-six of them didn’t answer any question and the remaining two didn’t answer a high percentage of questions, especially the key questions of the study - and 22 were made by people over 64 years old). The final response rate was 61,3% (325/530).

### Sampling error

Finally, the margin of error for the entire sample was ±5.56%, at a confidence level of 95.5% (2σ) and under the assumption of maximal uncertainty (p = q = 50).

### Data collection

To collect the data, we designed a Self-Administered Questionnaire, based on questions and answers that included different demographic, family, social, labor and economic variables. Also, we developed the instructions for self-administration of the questionnaire.

In order to develop a comprehensive questionnaire we conducted a pre-test with FM patients to examine the acceptance and comprehensibility of the questions. We requested to answer the questionnaire to 15 patients diagnosed of Fibromyalgia and enrolled in the Fibromyalgia Unit of Hospital Clínic. We analyzed and discussed with the patients: the time to answer the questionnaire, the comprehension of the instructions to complete the questionnaire and the unanswered questions or with the answer “I don’t know”. The answers given by the patients from the test were compared with the previous information available of each patient in order to verify the concordance with understanding of the questions. With this information we proceeded to make the pertinent changes to the questionnaire to be used for the study. Finally, the questionnaire consisted of 140 multiple-choice questions divided into 6 sections:

1. *Identification,* with the PHCC data and patient profile.
2. *Health,* covering characteristics of the disease (ages of symptoms onset and diagnosis, specialist who made the diagnosis, comorbidities, treatments), perceived health status, and sources used by patients to get information on the disease.
3. *Home and Family*, which included household characteristics, family life satisfaction, disease influence on relationships, roles and responsibilities, degree of dependence on family, economic situation, household income and expenditure, and strategies to offset the impact.
4. *Work or Employment*, including professional features and enterprise characteristics, employment history, current employment status, level of satisfaction, motivation and stress, task dynamics and characteristics, performance, sick leaves and labor absenteeism, relationships with colleagues and company, trade union membership, adaptations and supports, changes and strategies in terms of accessing and remaining at work, etc. …
5. *Supports, Acknowledgements and Resources*, which included assessment of family, employment, social and administrative support, recognition of permanent incapacity to work, recognition of disability pension, healthcare resources used, assessment and level of satisfaction with the resources, community resources to support patients, affiliation and assessment of patients’ associations, assessment of administrative responses related to his/her disease and order of priorities with regard to research, healthcare, benefits, etc. …
6. *Batteries or Complementary Questionnaires*: FM Impact Questionnaire (FIQ) [17], Health and Quality of Life Questionnaire (SF-36) [18] and Family Distress Questionnaire (APGAR) validated in Spanish [19].

Besides, there were other scales used, such as visual analog scales ranking from *0* to *10* cm to measure the degree of job satisfaction (with *0* as “not satisfied at all” and *10* “completely satisfied”), work motivation (with *0* as “not motivated at all” and *10* “completely motivated”), perceived stress at work (with *0* as “no perceived stress” and *10* “with maximal perceived stress”).

Also, it was measured the degree of impact perceived by patients in their life in general, family life and the changes generated in their relationships, roles and tasks related to FM, as well as in their work and professional life, where *0* corresponded to “nothing negative” and *10* to “strongly negative”.

Examples of questions from the Self-Administered Questionnaire in the three most significant aspects of the study are as follows:

- Family*. “If your FM has been the cause (mainly or partly) of some changes: how has been the impact that these changes have had on your family life? Mark with X the corresponding value (0 - not negative at all, 10 - completely negative). Changes to evaluate - through multiple responses - concerned patients’ dedication to housework, conducting family planning, social and leisure activities, couple relationships and effects on physical health of other family members”.*
- Employment*. “If the workplace and/or working conditions are adapted to your current capacity, would you rather work? The response options were No/Yes/It depends”.*
- Supports, Acknowledgements and Resources*. “Mark with X the extent to which it is considered to be important that the Public Administration provides the services to FM patients in the following fields. This multiple-choice question measured on the scale from 0 to 10 the following aspects: Labor/partnership/scientific/educational/pensions/health/awareness/social environments”.*

### Statistical analysis

The main analysis of the study was to describe the distribution of the different variables included through of frequency or statisticals analysis using the statistical package SPSS version 18. We have also made some comparisons specially between patients who were working at the time of the study versus those who were not. The difference in life-years worked or work satisfaction, Motivation and Job Stress scales between these patients were analyzed by t-tests for comparison of means and the difference in difficulties perceived to performed the work was analyzed by chi-square tests and the pairwise test of the equality of proportions (z-test) with the Bonferroni correction for multiple comparisons of proportions. Finally the relationship between the degree of satisfaction with healthcare professionals and the delay in receiving a diagnosis was analyzed by Pearson correlation coefficients.

This study was approved by the Clinical Research and Ethics Committee of *Hospital del Mar* of Barcelona. The inclusion of patients and data collection occurred between June 1, 2011 and April 10, 2012. After obtaining the informed consent from the patients, researchers in collaboration with the PHCCs handed to them the Self-Administered Questionnaire, along with the instruction manual. The patients completed the questionnaire at home and returned it completed a few days later.

## Results

### Identification

#### Sampling characteristics

The sampling obtained was of 325 patients (96.6% were women), with an average (standard deviation - sd) age of 51.9 (8), with% of marital status (married - 75%, singles - 10%, widows/widowers - 5%, separated/divorced - 9.6%) and with an educational level that includes 64% of patients with completed Secondary Education, 8% with Tertiary Education qualifications and 23% with University Degrees.

No differences were found in the territorial characteristics by regions or stratum population between the patients excluded and the patients who participated in the study.

### Health

The mean (sd) age when the first symptoms related to the disease occurred was 37 (11) years old, receiving a diagnosis with the mean (sd) time of 6.6 (8) years after the symptoms were first experienced, with an average (sd) age of 43 (9) of the patient at the time of diagnosis receiving. The mean (sd) time between the onset of the first symptoms of FM and the time the study was conducted was 15.5 (10) years.

Eighty-four percent of the patients reported suffering from a concomitant comorbidity, including 67% with other musculoskeletal diseases (especially musculoskeletal diseases of degenerative or mechanical nature), 35% with psychopathological disorders, 27% with digestive disorders, 23.5% with cardiovascular disorders and 19% with endocrine-metabolic diseases. Twenty-eight percent of FM patients reported having been diagnosed with Chronic Fatigue Syndrome.

#### Perceived health status

Only 5% of patients described their health as good or very good.

While analyzing the quality of life related to health status - through the SF-36 questionnaire - we observed a mean (sd) value of 28.2 (6.9) in Physical Health and 34.8 of Mental Health (13.3). The impact index of the disease, according to the FIQ, showed a mean (sd) number of 75.5 (15.5). These results are consistent with the responses of the patients in the self-administered questionnaire on the degree of perceived impact in different areas of life (Table 1).

**Table 1:** **Perceived impacts on different areas of life**

**Life Aspects**	Mean (sd) (0 to 10)*
**Life in General**	**7.8 (2.1)**
Health	8.7(2.0)
Work	8.5 (2.3)
Leisure, recreation and sports activities	8.0 (2.3)
Mental and emotional health	7.9 (2.5)
Career	7.2 (3.5)
Economy	6.9 (3.3)
Relationship with partner	6.6 (2.9)
Citizen rights	6.2 (3.8)
Family	5.9 (3.1)
Friendship	5.8 (3.2)

*Response scale from *0* to *10*, where *0* = not negative and *10* = totally negative.

### Work/Employment status

A total of 146 (45%) patients with FM were working at the time of the study and 156 (48%) had previously had an income-generated occupation. The total number of FM patients that answered the questionnaire had done paid work throughout their life, working, on average (sd), 23 (10) years, with few differences between the patients who were working at the time of the study *versus* those who were not, 24 (10) years and 20 (10) years, respectively (p: non significant).

Various professions which the patients had had before or had at the moment of the study were focused, especially, on the following sectors: Services (81%), Industry (12%), Construction (3.5%) and Agriculture (3.5%). While analyzing the work environments of the patients with FM, it was observed that 46% of them worked in the public sector, 30% in the private sector, 11.5% were self-employed, 3% were entrepreneurs and 9% other sectors.

#### Work satisfaction

The average (sd) of the Scale of Satisfaction, Motivation and Job Stress in FM patients was 6.5 (2.7), 6.1 (3) and 7.3 (2.9), respectively. No significant differences were observed among the patients who were working and those who had stopped working.

#### Employment status and situation

Seventy percent of the FM patients reported having many or enough difficulties while performing their work, executing common tasks, dealing with physical or environmental conditions and/or coping with the usual working hours. The frequency of these difficulties was higher for the patients who had stopped work than for those who were working (84% *versus* 65%, respectively) (p < 0.01).

**Figure 2:**
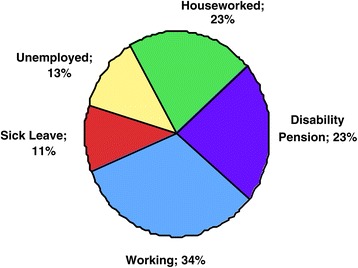
**Work status in FM patients (n: 325).** This figure describes the percentage of patients with Fibromyalgia in relation to its administrative status labor.

Finally, while analyzing the employment situation of FM patients at the time of the study, we observed that while 34% of the patients were working, 13% of the patients were unemployed, 11% were on sick leave, 23% were receiving a pension because of their inability to work (4% had inability to work partially and were working at the same time with other paid work) and 23% of the patients were doing housework without income-generated work (Figure 2). As a whole, 42% of the patients with FM were not in the labor force at the time of the study.

#### Workplace support

Sixty-three percent of patients had informed their work environment about their disease, and 30.4% had undertaken adjustments at work, which was 69% of the requested. Most of patients (70%) considered it as a positive change. 41% of patients had not requested adjustment changes though they considered that it might be appropriate and 15% did not consider it appropriate.

Nineteen percent of the patients changed their company or business and 66% of them are performing a different activity.

### Family consequences

It should be noted that 70% of FM patients live with a partner and 60% of respondents have children at home.

#### Family dysfunction

The degree of family dysfunction measured by the APGAR questionnaire showed that 69% of families maintained a Normal Family Life, 23% of patients experienced Mild/Moderate Family Dysfunction and 8% of respondents had Severe Family Dysfunction.

#### Family satisfaction

Twenty-three percent of patients reported being scarcely satisfied or not satisfied at all with their family life, and 59% had many difficulties in their relationships with their partner. Seventeen percent of patients are divorced and, in half of the cases, they believed that the disease could have influenced it. Sixty-seven percent of patients reported having many difficulties in their sexual relationships as a result of the disease.

#### Consequences in the home environment

Eighty-six percent of patients reported having enough or a lot of difficulties in doing household chores. Forty-four percent of respondents were fairly or totally dependent on a member of the family while performing household duties. In 56% of cases, a family member had to take over enough or many of the tasks previously performed by respondents and 27% of patients reported that a member of their family had had to change his/her normal work activity because of the patient’s difficulties.

Seventy-three percent of patients reported having enough or very difficult to perform leisure activities with their family and 69% also found it quite or very difficult to make family plans or projects, as a result of their disease.

#### Family support

If we look at the family support perceived by patients, it should be noted that 66% of patients reported that their family understood, helped and supported them in their fight against the disease, quite or completely. However, 45% of respondents said that their family did not understand the disease and that they did not follow the doctors’ recommendations. When analyzing the expectations, 98% of FM patients replied in the affirmative that the disease would affect their family and home environment in the future, and the need for being helped more with their household chores is the factor most related to this change.

#### Consequences on household economy

In 42.2% of households, the household economy depended, to a greater or lesser extent, on the income contributed by the person affected by FM, in 17.1% of the cases, exclusively.

65.6% of patients reported having difficulties with their household economy, and in 65% of the cases, household income had decreased, with an average (sd) of 708 (504) Euros per month. The extra expenses of 81% of patients had increased in relation to their disease, with an average (sd) of 230 (192) Euros per month. 31% of patients reported that this increase in their disease-related expenses made up more than 300 Euros per month. Table 2 shows a list of the main causes related to these expenses.

**Table 2:** **Major causes of extra expenses that FM patients experience**

**Extra expenses**	%*
Medication in drug stores	59.4
Physiotherapy	45.7
Gim/pool	41.0
Medical consultations	36.8
Dietary, herbal and homeopathic supplements	31.4
Home care support	29.8
Other therapies/treatments	26.7
FM Association dues	24.8
Transportation extra expenses	21.6
Home adaptations	19.4
Psychological therapy	18.1
Complimentary investigations	17.5
Other expenses	6.0

*Multiple items related to the extra expenses.

### Health, administrative and social support

The degree of satisfaction with the overall support received, perceived by patients regarding their disease in different environments was evaluated. It should be noted that 38% of FM patients reported that the National Health System is the environment that provides less support.

#### Health environment

Seventy-two percent of FM patients participated in the study reported are attending exclusively to Public Health Departments, and 28% of patients complimented it visiting a Private Healthcare Entity. The professionals that patients visit regularly are Family Physicians (91%), Rheumatologists (55%), Psychiatrists (29%), Psychologists (22%), Physiotherapists (22%), Doctors trained to treat FM (16%), Occupational Therapists (2%).

The overall satisfaction with healthcare professionals showed - up to the establishment of diagnosis and during treatment - was low, with a mean (sd) of 4.9 (2.9) out of 10 (where *0* = “not at all satisfied” and *10* = “extremely satisfied” with the medical care received), with family physicians being the highest rated. There was a negative relationship between the degree of satisfaction and the delay in receiving a diagnosis (r −0.20 p < 0.008).

#### Administrative support (*autonomous community, government, judiciary,* …)

Twenty-three percent of patients had achieved a degree of State recognition of Permanent Disability, which involves financial compensation. This situation was recognized administratively in half of the patients (11.5%); the other half (11.5%) required court action, upon the existence of a dispute with the Administrative Institutions of Government.

At the time of the study, 8% of FM patients were carrying out administrative or judicial formalities requiring recognition of their Permanent Disability.

#### Social organizations *(patient association, trade unions,…)*

At the time of the study, 26% of patients were members of specific FM Associations. The principal reasons to become a member were as follows: get guidance/advice (45%), learn more about their disease (27%), feel supported and understood (24%) and carry out common activities or obtain cheaper services (18%). Fifty-nine percent of the association members were participating in the organized activities. The degree of satisfaction with the association was high (mean of 0–10 (sd): 7 (3.1)). Only 20% of patients did not feel satisfied.

#### Patient priorities

Referring to the priorities that Public Administrations should take into consideration while developing health and social policy, the patients participated in the study gave high priorities to the areas of Scientific Research (87%), Healthcare (77%), Pensions (66%), Work Environment (56%), Education (35%), Awareness and Social Resources (30%) and Association environment (23%).

## Discussion

This study shows a significant influence of FM on family and work environment with almost half of patients having lost their capacity for work*.*

At the time of the study, only 34% of patients were actively working, 23% were in an uncertain situation reorganizing their activities (11% on sick leave due to the disease and 13% unemployed due to losing their job), 23% had redirected their employment activities into housework and the remaining 23% had obtained a disability pension for recognized incapacity for work status.

These data confirm the reports published in other countries [4-7] which showed that labor force participation of FM patients was greatly affected and it is established a more representative measure of this impact in our country, after variability published by previous studies in this area [9-12].

Sick leaves episodes are frequent in FM patients. The longitudinal study conducted in a PHCCs by Sicras-Mainar et al. [12] showed that FM patients experiences an average of 21 days of work lost due to this problem. Our study shows that half of the patients had sick leave episodes over the last year, especially all those patients that finally lost their ability to work. This fact should be noted in order to establish therapeutic strategies relevant to the work activity in these patients. In our study, 63% of patients had reported the existence of the disease in their workplace and only 30% of them had work adaptations.

This study also shows the significant change that occurs in the dynamics of FM patients’ characterized by the functions changing mainly associated with disability. Forty-four percent of patients reported to be dependent on a family member in terms of household chores. In 27% of the FM relatives, a main member of the family had to change his/her normal work activity.

There are no published studies analyzing the actual burden carried by relatives of FM patients, although some published experiences have demonstrated that FM patients need more help in their household activities, which changes the family functions increasing workloads and responsibilities of other family members [20,21]. These changes are not only determinants of the family dynamics, but also the degree of family satisfaction. In this sense, 23% of patients reported low levels of satisfaction with their family life and 59% reported many difficulties in the relationship with their partner, the facts referred to in few studies in this area [8,22].

Marcus et al. [8] in a recently conducted online survey of a large number of patients, using the Relationship Assessment Scale (RAS) - a validated scale to measure relationship satisfaction [23] - noted that half of the participants reported that FM affected in a mild-to-moderate way their relationship with their partner and half of them valued as unsatisfactory their relationship with their current partner. The authors of this study defined the lack of relationship satisfaction on a 7-point scale, with a RAS average being less than 4. What is also notable in this study [8] is the observation that satisfaction was affected by the presence of mood disorders and a higher degree of FM severity.

In our work, in addition to 23% of patients who reported being little or not at all satisfied, 29% said to be moderately satisfied, with a possibility that some of the latter might have had low levels at a RAS-scale, which makes concordant the results of both studies.

Reich et al. [22] found that family satisfaction was negatively related to pain and physical function. Patients with more pain and severe disability had higher burdens on caregivers and a lower perception of family support related to family dissatisfaction, especially when there were high levels of uncertainty concerning the disease. This situation does not occur with other patients suffering from other conditions such as osteoarthritis chronic pain, where the level of uncertainty is lower. In our study, 45% of patients report that their partner or children do not understand their disease.

The fact that the family dissatisfaction might be related to increased burdens of family caregivers, low perceived support and high levels of uncertainty suggests that any intervention in order to reduce burden, increase the perception of family support or reduce uncertainty should be integrated into therapeutic programs [24].

Other issues that may interfere in family relationships are alterations of mood and health status that occur in patients and families. Some studies [25,26] have reported a worse health among couples and friends of patients with FM than those relatives of healthy controls, although they have not been corroborated by other authors [27]. In our study, 58% of patients report that FM has greatly (23.4%) or somewhat (39%) affected the state of mind of some family members, but it has not been corroborated by a direct evaluation.

We must not forget the importance of the relationship and the malfunctions that occur during sexual intercourse. Sixty-seven percent of the patients reported many sexual difficulties as a result of the disease. In addition to the difficulties associated with pain or libido impairment, the disease impact on the couple is also influenced by the decrease in family satisfaction, with all of this being predictable in terms of involvement in sexual relationships of these patients [28].

Finally, we would like to remark that the employment impact and changes in the family dynamics that occur in this disease result in large economic losses. Two studies on economic costs associated with the disease carried out in our country [29,30] are consistent with the results published in other countries [31] and show that the cost of a FM patient represents between 8,000 and 10,000 Euros/year per patient, with 70% of the costs associated with labor impact in terms of loss of employment, sick leaves, disability compensation, etc. …

Our study analyzes the economic consequences through the loss of income and extra expenses in FM relatives regardless of the costs incurred by the Health Public System, State Administration or Insurance and Business companies. Even so, in 65% of the households of FM patients, revenues decreased a mean (sd) of 708 (504) Euros/month in relation to the presence of the disease and, in 81% of the households, the extra expenses increased a mean (sd) 230 (192) Euros/month in relation to the disease. This might reveal financial expense greater than previously estimated.

The major strength of this study is the sample of FM patients well distributed throughout the Spanish territory who attend regularly PHCCs. Sample patients of specialist clinics would have been a possible alternative, especially to improve the response rate, but there would be a significant selection bias. The response rate was 61,3% due to certain number of PHCCs did not agree to participate in the study. The analysis of non-participating centers showed a slightly higher proportion located in smaller municipalities of non-urban areas. While there may be some small differences between areas that affect the characteristics of the sample, we have no data to suggest the existence of significant differences in terms of universality and using the Public Health System by FM patients in these territories.

The final response rate has been associated with a number of patients excluded as a result of a significant deficit in the questionnaire responses (twenty-six patients didn’t answer any question and the remaining two didn’t answer a high percentage of questions, especially the key questions of the study), or being outside the age range required for the study. No differences were found in the territorial characteristics by regions or strata between the patients excluded and the patients who participated in the study.

It should be emphasized that patients included in this study are a sample of patients presented to PHCCs with impaired state of health, as evidenced by scores on the FIQ and SF-36 questionnaires, therefore, the study can not take into account patients that interact less frequently with the Public Health System, being less severely affected. This limitation is insurmountable, but we choose to study a sample in this area as a less unfavorable option, and avoid studying patients selected at specialized clinics, hospitals or patients’ associations, which might bring together patients with higher socio-professional impact.

## Conclusions

People with FM who have visited PHCCs in Spain experience severe consequences on family environment, especially related to the patient’s functional limitations, the economic status… On the other hand, the data support the fact that the disease is often associated with an employment status change and a loss of ability to work. Longitudinal studies are needed to demonstrate the evolution of these consequences and its modification while treated.

## Abbreviations

FM: Fibromyalgia
Sd: Standard deviation
PHCC: Primary Health Care Center
AC: Autonomous Communities
FIQ: FM Impact Questionnaire
RAS: Relationship Assessment Scale
SF-36: Short Form 36
APGAR: Adaptability, Partnership, Growth, Affection, Resolve
